# Hyaluronic-Acid-Tagged Cubosomes Deliver Cytotoxics Specifically to CD44-Positive Cancer Cells

**DOI:** 10.1021/acs.molpharmaceut.2c00439

**Published:** 2022-08-08

**Authors:** Arindam Pramanik, Zexi Xu, Nicola Ingram, Patricia Louise Coletta, Paul A Millner, Arwen I I Tyler, Thomas A Hughes

**Affiliations:** † School of Medicine, University of Leeds, Leeds LS2 9JT, United Kingdom; ‡ School of Food Science and Nutrition, University of Leeds, Leeds LS2 9JT, United Kingdom; § School of Biomedical Sciences, University of Leeds, Leeds LS2 9JT, United Kingdom

**Keywords:** cubosomes, CD44 receptor, hyaluronic acid, liquid crystalline lipid nanoparticle, tumor spheroids

## Abstract

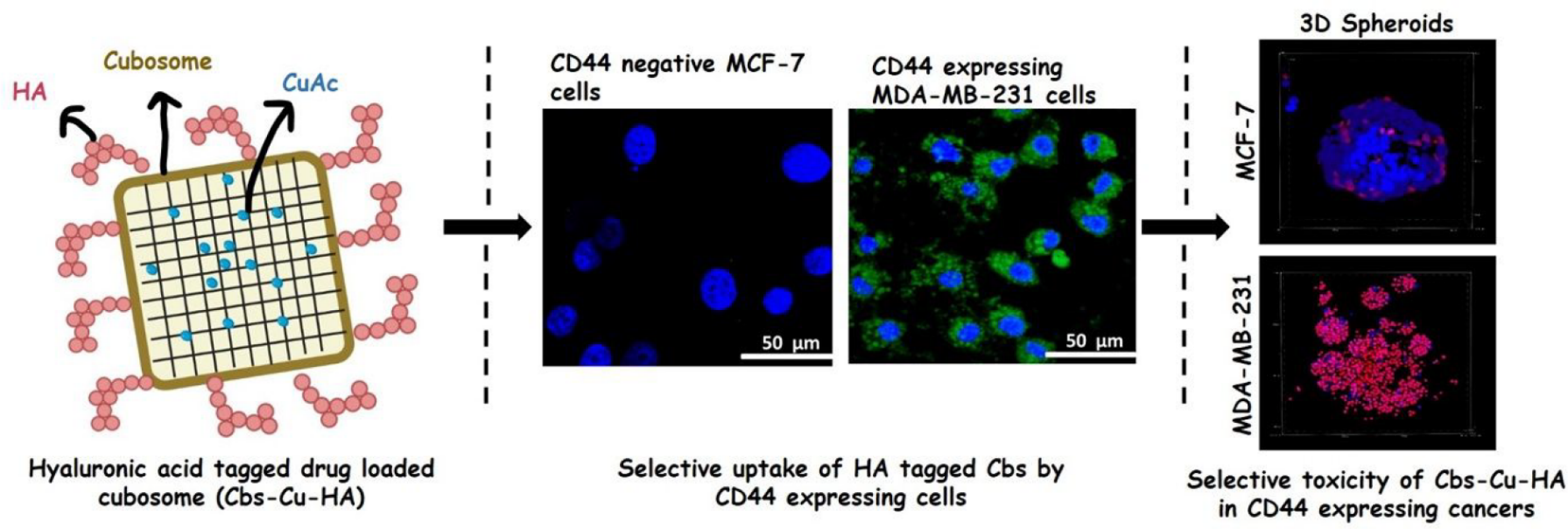

Delivery of chemotherapy drugs specifically to cancer cells raises local drug doses in tumors and therefore kills more cancer cells while reducing side effects in other tissues, thereby improving oncological and quality of life outcomes. Cubosomes, liquid crystalline lipid nanoparticles, are potential vehicles for delivery of chemotherapy drugs, presenting the advantages of biocompatibility, stable encapsulation, and high drug loading of hydrophobic or hydrophilic drugs. However, active targeting of drug-loaded cubosomes to cancer cells, as opposed to passive accumulation, remains relatively underexplored. We formulated and characterized cubosomes loaded with potential cancer drug copper acetylacetonate and functionalized their surfaces using click chemistry coupling with hyaluronic acid (HA), the ligand for the cell surface receptor CD44. CD44 is overexpressed in many cancer types including breast and colorectal. HA-tagged, copper-acetylacetonate-loaded cubosomes have an average hydrodynamic diameter of 152 nm, with an internal nanostructure based on the space group Im3m. These cubosomes were efficiently taken up by two CD44-expressing cancer cell lines (MDA-MB-231 and HT29, representing breast and colon cancer) but not by two CD44-negative cell lines (MCF-7 breast cancer and HEK-293 kidney cells). HA-tagged cubosomes caused significantly more cell death than untargeted cubosomes in the CD44-positive cells, demonstrating the value of the targeting. CD44-negative cells were equally relatively resistant to both, demonstrating the specificity of the targeting. Cell death was characterized as apoptotic. Specific targeting and cell death were evident in both 2D culture and 3D spheroids. We conclude that HA-tagged, copper-acetylacetonate-loaded cubosomes show great potential as an effective therapeutic for selective targeting of CD44-expressing tumors.

## Introduction

In 2020, almost 10 million cancer-related deaths were recorded globally, of which colorectal and breast cancer accounted for ∼0.9 and ∼0.6 million, respectively.^1^ Management for the majority of breast and colorectal cancer cases includes systemic cytotoxic chemotherapy at some point in the treatment pathway, but unfortunately, chemotherapy resistance of cancer cells is a common problem as reflected in cancer recurrences or disease progression after therapy that lead to cancer deaths.^2,3^ Doses of systemic chemotherapy in patients are limited by side effects caused by their influence on off-target tissues. Consequently, research has focused on enhancing chemotherapy delivery specifically to cancer cells, with the aims of increasing local doses to kill more cancer cells while reducing off-target side effects. An increasing number of chemotherapy nanomedicines have now been approved for use in therapy; these typically encapsulate chemotherapy agents in nanosized particles to aid delivery to cancer cells through passive accumulation within tumors, which results from the enhanced permeability and retention effect, and through increased bioavailability.

Lipid lyotropic liquid crystalline nanoparticles with an internal cubic phase nanostructure, referred to as cubosomes, have gained recent attention as potential chemotherapy delivery agents.^4,5^ Cubosomes present numerous advantages over liposomes, the most-commonly approved nanocarrier, including improved stability, high drug encapsulation efficiency, and an ability to encapsulate both hydrophobic and hydrophilic drugs.^6^ A further development of nanocarriers is that they can be actively targeted to cancers by functionalizing their surface to promote specific binding to cancer cells. We have previously successfully used antibodies against cancer cell antigens for targeting nanoparticles to cancers,^7^ and we have also recently demonstrated active targeting of cubosomes to cancer cells.^8^ In that work, we used drug-loaded cubosomes targeted with affimers against the cancer cell antigen CEA to direct cancer therapy in preclinical models of colorectal cancer.^8^ The choice of the cancer cell antigen against which to direct these therapies is a key factor, as this defines the range of cancers that could potentially be treated. Others have targeted cubosomes to cancer cells using biotin,^9^ folic acid,^10^ and antibodies for the epidermal growth factor receptor.^11^ The CD44 receptor is a further potential target for nanoparticle delivery, by attaching its natural ligand hyaluronic acid (HA) to the surface of particles. There are several reports of use of HA as a targeting agent to deliver therapeutics^12,13^ or miRNAs^14^ to CD44-expressing tumors. CD44 itself is often overexpressed in various cancer types including both breast^15^ and colorectal.^16^ Of particular interest is that this overexpression is most prominent in the cancer stem-like cells within individual tumors;^17^ these cells are strongly associated with drug resistance and metastases and therefore represent the most important tumor cell population to target therapeutically. Therefore, CD44 is not only frequently overexpressed in a range of common cancers, but it is most highly expressed within the subpopulation of cancer cells within each tumor that is it most critical to target.

**Figure 1:**
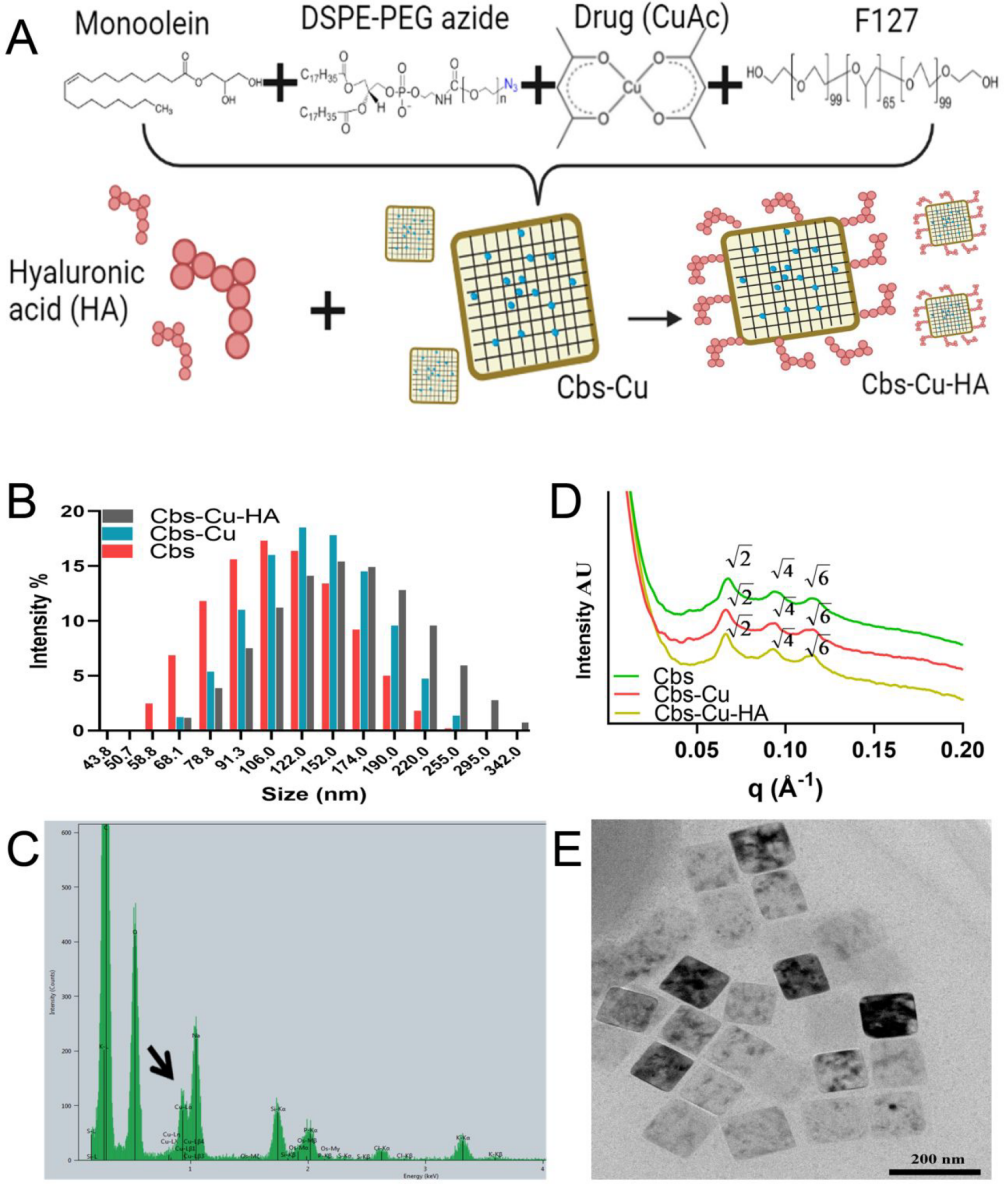
Characterization of cubosomes. (A) Schematic representation of Cbs-Cu-HA design. (B) DLS data showing the hydrodynamic diameter of Cbs, Cbs-Cu, and Cbs-Cu-HA. (C) EDAX spectrum of Cbs-Cu-HA with a black arrow indicating the peak representing copper. (D) SAXS patterns of Cbs, Cbs-Cu, and Cbs-Cu-HA at 37 °C. (E) TEM image of Cbs-Cu-HA.

In this study, we have developed and characterized a novel formulation of monoolein-based cubosomes functionalized with HA and loaded with the model anticancer drug copper acetylacetonate (see flow-scheme in Figure 1A). We have evaluated the specificity of this formulation for CD44-expressing cancers and have demonstrated effective CD44-dependent cytotoxicity in both breast and colorectal cancer cells. We conclude that HA-functionalized cubosomes show great potential as cancer therapeutics.

## Materials and Methods

### Small-Angle X-ray Scattering Clickable Cubosome Preparation and Drug Encapsulation

Cithrol GMO HP (MO) was gifted from Croda (Croda Personal Care, Goole, UK). It is a commercial version of monoolein containing a minimum of 92% monoester and a maximum of 8% diester. DSPE-PEG-2000 azide (DPZ) and 1,2-dioleoyl-*sn*-glycero-3-phosphoethanolamine-*N*-(7-nitro-2–1,3-benzoxadiazol-4-yl) (ammonium salt) (NBD-PE) were purchased from Avanti Polar Lipids (AL, USA), and Pluronic F-127 was purchased from Sigma-Aldrich (Gillingham, U.K.). Cubosomes were prepared by codissolving MO and DPZ in chloroform (Merck, New Jersey, USA) and mixing at an appropriate ratio (9–9.5 MO weight ratio to DPZ) in a glass vial and then evaporating the chloroform under nitrogen gas in a fume hood. To ensure complete drying and evaporation of the chloroform, the glass vials were put in a desiccator overnight at room temperature to obtain dry lipid films. Post drying, the lipid film was hydrated with phosphate-buffered saline (PBS; Sigma-Aldrich Gillingham, U.K) containing Pluronic F-127 between 2–7 wt % to the MO. Homogenous cubosome nanoparticle dispersions were prepared by tip sonicating the sample in 1 mL of buffer using a Q125 sonicator (Qsonica, USA) for 30 min in pulse mode (1 s pulse on, 1 s off) at 80% amplitude in an ice bath. The cubosomes were then passed through a mini extruder (Avanti Polar Lipids, USA) containing a polycarbonate membrane (Whatman, USA) of 100 nm pore size for size uniformity. For encapsulating drug in the cubosomes (Cbs-Cu), copper acetylacetonate (CuAc; Merck, USA) was dissolved in chloroform and added in various weight percentages (1–5% w/w) to the codissolved lipid mixtures before the nitrogen gas drying process, and the same process as detailed above was followed for the synthesis of Cbs-Cu. For removal of unencapsulated CuAc from Cbs-Cu, the sample was placed in Slide-A-Lyzer cassettes (2K MWCO, Thermo Scientific, UK) in PBS at 25 °C over a magnetic stirrer to perform dialysis. For the *in vitro* localization study, 0.5% w/w of the fluorescent lipid 1,2-dioleoyl-*sn*-glycero-3-phosphoethanolamine-*N*-(7-nitro-2–1,3-benzoxadiazol-4-yl) (ammonium salt) (18:1-NBD PE; Avanti Polar Lipids, USA) was codissolved with the MO & DPZ lipid mixtures in chloroform before the drying step.

Inductively coupled plasma optical emission spectrometry (iCAP 7600 ICP-OES Analyzer, Thermo Scientific, UK) equipped with a 240-position Cetac autosampler was used to estimate CuAc encapsulation in the cubosomes. Known concentrations of copper solutions were used as a standard curve for reference. The encapsulation efficiency (%) was calculated using eq 1
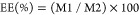
1where M1 represents the weight of drug encapsulated in mg (obtained from ICP-OES) and M2 represents total drug added (mg) to the cubosomes.

### Conjugation of Hyaluronic Acid with a Clickable Cubosome

Hyaluronic acid (HA; Sigma Alrich, USA; molecular weight 8–15 kDa) was attached to the cubosomes by click chemistry coupling using DBCO-PEG4-amine (Kerafast, Inc. Boston, USA). Briefly, 2 mL of (5 mg/mL) HA in RNase free water was treated with 1 mL of (0.5 mg/mL) of 1-ethyl-3-(3-(dimethylamino)propyl)carbodiimide (EDC; Sigma-Aldrich, USA) in RNase free water and 500 μL of (0.2 mg/mL) *N*-hydroxysuccinimide (NHS) in a 15 mL Falcon tube and rotated on a shaker for 12 h at room temperature. Post activation of the carboxylic group in HA, 1 mL of (2 mg/mL) DBCO-PEG4-amine in DMSO was added to the reaction mixture and incubated for 24 h at room temperature. The final product was dialyzed in 1× PBS using Slide-A-Lyzer Dialysis Cassettes, 2K MWCO (Thermo Scientific, Waltham, USA) to remove any unconjugated DBCO-PEG4-amine. The final DBCO-HA was freeze-dried.

For conjugation of HA on Cbs-Cu (Cbs-Cu-HA), 1 mg of DBCO-HA was added to 5 mL of a (10 mg/mL) homogeneous solution of Cbs-Cu in PBS, and the sample was left under magnetic stirring at 250 rpm for 4 h at room temperature. The conjugated Cbs-Cu-HA was then dialyzed using Slide-A-Lyzer Dialysis Cassettes, 7K MWCO (Thermo Scientific, Waltham, USA) to remove unconjugated DBCO-HA. FTIR spectroscopy (Platinum ATR, Model −Alpha, Bruker, UK) was used to confirm the covalent conjugation between the azide group of cubosomes and the DBCO group attached to the HA (Figure S1).

Small-angle X-ray scattering (SAXS) was used to study the internal nanostructures of the Cbs, Cbs-Cu, and Cbs-Cu-HA at 37 °C (5 min equilibration and accuracy of ±0.1 °C). Synchrotron SAXS measurements were carried out on beamline I22 at the Diamond Light source. The synchrotron beam was tuned to a wavelength of 0.69 Å with a sample to detector distance of 3.7 m, and the 2D SAXS patterns were recorded on a Pilatus 2 M detector. SAXS experiments were also conducted on a lab-based Xeuss 3.0 (Xenocs, France) beamline equipped with a liquid gallium MetalJet X-ray source (Excillum, Sweden), which has an energy of 9.2 keV, corresponding to a wavelength of 1.34 Å. 2D SAXS patterns were recorded on an Eiger2 R 1 M detector (Dectris, Switzerland), and the sample to detector distance was set to 0.8 m, giving a *q* range of 0.01–0.4 Å^–1^. Silver behenate (*a* = 58.38 Å) was used to calibrate the SAXS data. SAXS images were analyzed using the IDL-based AXcess software package or the DAWN software.^18,19^

### Particle Size and Zeta Potential Measurements by Dynamic Light Scattering (DLS)

The hydrodynamic particle sizes of the three samples (Cbs, Cbs-Cu, and Cbs-Cu-HA) were measured using a Zetasizer Nano ZS90 (Malvern Panalytical, Malvern, UK) at a fixed backscattering angle of 173° at 25 °C. The refractive index of the cubosomes was set to 1.46 (pure MO) with an absorbance of 0.10. The refractive index of the dispersant (PBS) was set to 1.332 with a viscosity of 0.9053 cP. Aliquots of 100 μL of Cbs, Cbs-Cu, and Cbs-Cu-HA samples were added into 900 μL of PBS, and measurements were recorded. The instrument equilibration time was set for 120 s at 25 °C, and samples were run for 10 cycles with 10 measurements in each cycle. For zeta potential measurements, 100 μL of Cbs-Cu-HA was added to 900 μL of Millipore water (with a resistivity of 18.2 MΩ·cm at 25 °C) in a disposable zeta cuvette and was equilibrated for 120 s at 25 °C. The instrument was set to run 20 cycles with 10 measurements in each cycle.

### Transmission Electron Microscopy (TEM)

A transmission electron microscope (FEI Tecnai TF20) fitted with a field emission gun TEM/STEM along with an HAADF detector was used to study the size and morphology of Cbs-Cu-HA. A 10 μL aliquot of Cbs-Cu-HA (10 mg/mL) in PBS was added on a nickel grid coated with 200 mesh carbon film (EM Resolutions, UK), and any excess droplets were soaked up using an absorbent filter paper. The grid was left in a desiccator to dry for 24 h. The sample was imaged at 13 000× magnification at an accelerating voltage of 300 kV. The image was captured using a Gatan Orius SC600A CCD camera. Images were analyzed using Fiji ImageJ software (NIH, USA). Cbs and Cbs-Cu-HA samples were analyzed by an energy-dispersive X-ray equipped in the FEI Tecnai TF20 (Oxford Instruments INCA 350 EDX system/80 mm X-Max SDD detector) to confirm the encapsulation of CuAc in the cubosome (elemental copper as an indicator). The advantage of using a nickel grid over a standard copper grid in this study was to eliminate any background noise of copper during this EDX study.

### Cell Culture

MDA-MB-231, HT-29, MCF-7, and HEK-293 cells were originally obtained from the ATCC and were subjected to mycoplasma testing and STR typing (Source Bioscience, UK) before use. Cells were grown in DMEM (Thermo Scientific, Waltham, USA) growth medium supplemented with 10% (v/v) fetal calf serum (FCS; Thermo Scientific, Waltham, USA) and penicillin/streptomycin (Thermo Scientific, Waltham, MA, USA) at 100 units/mL. All cells were cultured in a humidified incubator with 5% CO_2_ at 37 °C. Cells were maintained and experiments were conducted at cell densities that allowed exponential growth.

### Immunofluorescence and Cubosome Localization

Cells were grown on coverslips with complete growth medium for 48 h and then washed in PBS and fixed with 4% (w/v) paraformaldehyde (Merck, New Jersey, USA) in PBS at room temperature for 10 min. The fixed cells were further washed with PBS and permeablized with 0.2% (v/v) Triton X-100 (Merck, New Jersey, USA) in PBS in an ice bath for 10 min. Cells were then washed with PBS several times and blocked with 5% (v/v) FCS in PBS for 1 h in an ice bath. Cells were then incubated with mouse IgG1 antihuman CD44 monoclonal antibodies (catalogue 5640, Cell Signaling Technology, USA) at a 1:1600 dilution overnight at 4 °C. The following day, several washes were performed with wash buffer, comprising 0.5% (v/v) FCS and 0.05% (v/v) Tween-20 in PBS. Cells were then incubated with AlexaFluor 594 labeled antimouse IgG1 antibodies (catalogue A-11032, Thermo Scientific, USA) at 1.5 μg/mL for 1 h at room temperature in the dark. Cells were then washed with wash buffer several times and mounted with Fluoromount-G mounting media with DAPI (Thermo Scientific, USA) before analysis using confocal microscopy (Nikon A1R; DAPI: 405 nm laser, 407 nm excitation filter, 450 nm emission filter; AlexaFluor 594: 590 nm laser, excitation filter 590 nm, emission filter 617 nm). Images were captured using a 100× objective with a numerical aperture of 1.4. The images were analyzed using the NIS-element viewer software (v5.20.01). For cubosome localization studies, MDA-MB-231 and MCF-7 cells were seeded in glass-coated chamber slides (Thermo Scientific, USA) overnight for 18 h. Cells were then treated with 20 μg/mL of Cbs-NBD with and without HA tagging for 24 h. Cells were gently washed with PBS and incubated with 5 μg/mL of Hoechst 33342 for 15 min before the cells were imaged using confocal microscopy (100× objective; numerical aperture 1.4). Hoechst 33342 was imaged using a 405 nm laser, with excitation and emission wavelengths of 407 and 450 nm; NBD was imaged using a 488 nm laser, with excitation and emission wavelengths of 488 and 525 nm, respectively. Images were captured using Galvano scanning mode and analyzed using the NIS-element software (v5.20.01).

### Cell Survival and Apoptosis Assays in 2D Culture

MDA-MB-231, MCF-7, HT-29, and HEK-293 cells were seeded in 24 well culture plates in growth media at densities of 2.5 × 10^4^ cells/well and incubated overnight for 18 h. Cells were then treated with concentrations ranging from 0 to 125 μg/mL of Cbs, Cbs-Cu, and Cbs-Cu-HA for up to 24 h. Post treatment, MTT assays were performed as detailed in our previous work.^8^ Apoptosis was assessed using annexin V/propidium iodide assays and flow cytometry. Briefly, MDA-MB-231 and MCF-7 cells were treated with 75 μg/mL of Cbs-Cu-HA for time points up to 48 h. Post treatment, cells were washed with annexin binding buffer, and annexin V-FITC (Thermo Fisher Scientific, Waltham, MA, USA) was added to the cells at a final concentration of 2 μg/mL and incubated for 15 min under dark conditions. Immediately prior to flow cytometry, 1 μg/mL of propidium iodide (Thermo Fisher Scientific, Waltham, MA, USA) was added, and cells were analyzed using a CytoFLEXS flow cytometer (Beckman Coulter, UK). Data were analyzed on FlowJo software v10.6.1.

### Cell Survival and Apoptosis Assays in 3D Tumor Spheroids

To create spheroids, low adherent round-bottom 96 well plates were used. MCF-7 and MDA-MB-231 cells (1000/well) were added with 250 μL of DMEM containing 10% (v/v) FCS along with 2.5% Matrigel (Corning, New York, USA). The 96 well plates were then centrifuged for 10 min at 360*g* and then incubated for 48 h for the formation of spheroids. The spheroids were then treated with 75 μg/mL of Cbs-Cu-HA for up to 24 h. Next, cellular viability within spheroids was quantified: spheroids were treated with Hoechst 33342 (5 μg/mL) for 30 min and propidium iodide (1.5 μg/mL) for 10 min. Red fluorescence (positive staining with propidium, signifying nonviable cells) and blue fluorescence (positive staining for Hoechst 33342, which permeates both viable and nonviable cells) were quantified using ImageJ software (NIH, USA), and blue:red ratios were used to calculate spheroid survival. Apoptosis was assessed by quantifying caspase 3 cleavage by Western blots. Spheroids were lysed using a mild mechanical vortex at regular intervals in the lysis buffer containing 1% Triton X-100, 50 mM Tris (pH 7.5), 10 mM EDTA, 0.02% NaN_3_, and a protease inhibitor mixture (Roche Diagnostics, Germany). Total protein concentration was measured using the Bradford method, equal amount of protein was loaded in 4–12% precast polyacrylamide gel (Bio Rad, California, USA), and electrophoresis was performed for 90 min at 120 V. The proteins were then transferred to a PVDF membrane and blocked with 5% (w/v) nonfat skimmed milk in TBST (Tris-buffered saline with 0.1% Tween-20) for 1 h. The membrane postblocking was labeled with caspase 3 primary antibody (Cell Signaling Technology, USA) as an apoptosis protein marker, and β-actin antibody as a loading control (Cell Signaling Technology, USA) was used. Next, HRP-tagged secondary antibodies (Cell Signaling Technology, USA) were added, and Pierce ECL reagents were used to visualize the target proteins bands using a ChemiDoc instrument (Biorad, USA). Densitometry was performed by quantifying the band intensities using ImageJ software. The caspase 3 intensity recorded was plotted relative to intensity of β-actin.

### *In Vivo* Experiments

Female BALB/c nude mice, aged 6 weeks, each weighing approximately 20 g, were used for the *in vivo* study. All experiments were performed following local ethical approval and in accordance with the UK Animals (Scientific Procedures) Act 1986. Mice were housed in individually ventilated cages with a 12 h day/night cycle with provisions for ad libitum food and water. At the end of each experiment, mice were euthanized following standard procedures. A total of 12 mice were randomly divided into 2 groups (6 mice in each group). One group received intravenous (IV) injections of 100 μL of Cbs-Cu in PBS from a stock concentration of 18 mg/mL. The other group received 100 μL of saline and served as the control group. The IV administration was repeated thrice with 2 day intervals. The weight of the mice was monitored in both the groups during the experiment. After a further 5 days, mice were sacrificed, and organs (liver, kidney, heart, lung, brain, spleen) were studied for any necrosis or abnormalities. Tissue histology was conducted using hematoxylin and eosin staining and studied under the bright field microscope (Nikon Eclipse E1000).

## Results and Discussion

### Characterization of Cubosomes

Monoolein (MO)-based cubosomes were formulated and stabilized using DSPE-PEG2000-azide (DPZ) and Pluronic F127 as previously.^8^ DSPE-PEG2000-azide here serves an added advantage of enabling cubosomes to be functionalized with external-facing ligands using copper free click chemistry with the azide group. One of the challenges while formulating nanoparticles is controlling particle size, aiming for 100–200 nm diameters,^20−23^ which can penetrate the tumor vasculature to deliver drugs to diseases sites while avoiding rapid elimination.^24^ Various different ratios of MO, DPZ, and F127 were studied as detailed in our previous work;^8^ here we used MO:DPZ:F127 = 88.79:4.67:6.54 (w/w), which yielded a *Z*-average size of 106 nm for cubosomes without an external ligand or cytotoxic drug payload (“Cbs” Figure 1B; Figure S2); the polydispersity index (PDI) for these was 0.155 (Figure S2), indicating stable, mainly monodispersed particles.

Metal-based complexes of ruthenium, titanium, and platinum have successfully entered clinical trials as cancer therapeutics, and this has led to other new metallic complexes being studied as therapeutics.^25^ In this work, we have further extended our previous successful use of the copper complex copper acetylacetonate (CuAc) as a model drug.^8^ Use of copper compounds in cancer therapy is an area of intensive and expanding research, and copper has been shown to target a wide range of cancer-relevant molecular pathways.^26^ Furthermore, simple copper compounds such as CuAc are affordable in low resource settings; therefore, we expect our work could be more relevant in global terms, since the current first line cancer drugs used in the many developed countries are often unaffordable elsewhere.^27^

CuAc was encapsulated into cubosomes at 5% (with respect to MO), and this was confirmed by inductively coupled plasma optical emission spectrometry (ICPOES). For this study, elemental copper (Cu) was used as the marker for detection of CuAc. CuAc encapsulation of 82% was noted,^8^ which was in a similar range to a previous report for encapsulation of a photosensitizer into cubosomes.^28^ Inclusion of CuAc caused an increase in cubosome size to 125 nm (“Cbs-Cu” Figure 1B, Figure S2). Finally, the HA ligand was covalently attached to the external surface of the cubosomes, creating Cbs-Cu-HA nanoparticles, with a *Z*-average size of 152 nm (Figure 1B, Figure S2). FTIR spectroscopy was used to demonstrate loss of essentially all detectable azide groups on the cubosomes, showing that all potential HA-conjugation sites had been used (Figure S1). Polydispersity indexes for these particles remained good, with values of 0.159 and 0.131 respectively (Figure S2). Encapsulation of CuAc was further confirmed by energy-dispersive X-ray spectroscopy (EDAX), where characteristic Lα and Lβ peaks for copper (920–950 eV) can be observed in Cbs-Cu-HA particles (Figure 1C) as compared to bare cubosomes, Cbs (Figure S3).

The internal nanostructures of cubosomes at each step, i.e., bare (Cbs), drug encapsulated (Cbs-Cu), and HA-tagged (Cbs-Cu-HA), were studied using small-angle X-ray scattering (SAXS) at 37 °C (Figure 1D). All the samples at these two temperatures showed the presence of Bragg peaks in the ratio of √2:√4:√6 (corresponding Miller indices (hkl) 110, 200, 211), which index as a primitive bicontinuous cubic space belonging to space group Im3m. The lattice parameters of Cbs, Cbs-Cu, and Cbs-Cu-HA at 37 °C were 133.3, 135.1, and 137 Å, respectively. The addition of 5% CuAc and HA did not alter the phase transition but slightly increased the lattice parameters, as the bulky metal organic complex reduced the degree of monolayer spontaneous inverse curvature.

In context of nanoparticles, it has been observed that zeta values above ±40 mV correspond to high electrostatic stability;^29^ in our case, the zeta potential of Cbs-Cu-HA was −40.8 mV (Figure S4), which correlates well with our observation of extended particle stability (>21 days). Transmission electron microscopy (TEM) images of HA-tagged drug encapsulated cubosomes (Cbs-Cu-HA; Figure 1E) and of cubosomes without drug or HA (Cbs; Figure S5) show cubical structures, and the sizes were found in a similar range as the hydrodynamic diameters examined by the DLS.

### Hyaluronic Acid Effectively Targets Cubosomes to CD44-Expressing Cancer Cells

**Figure 2:**
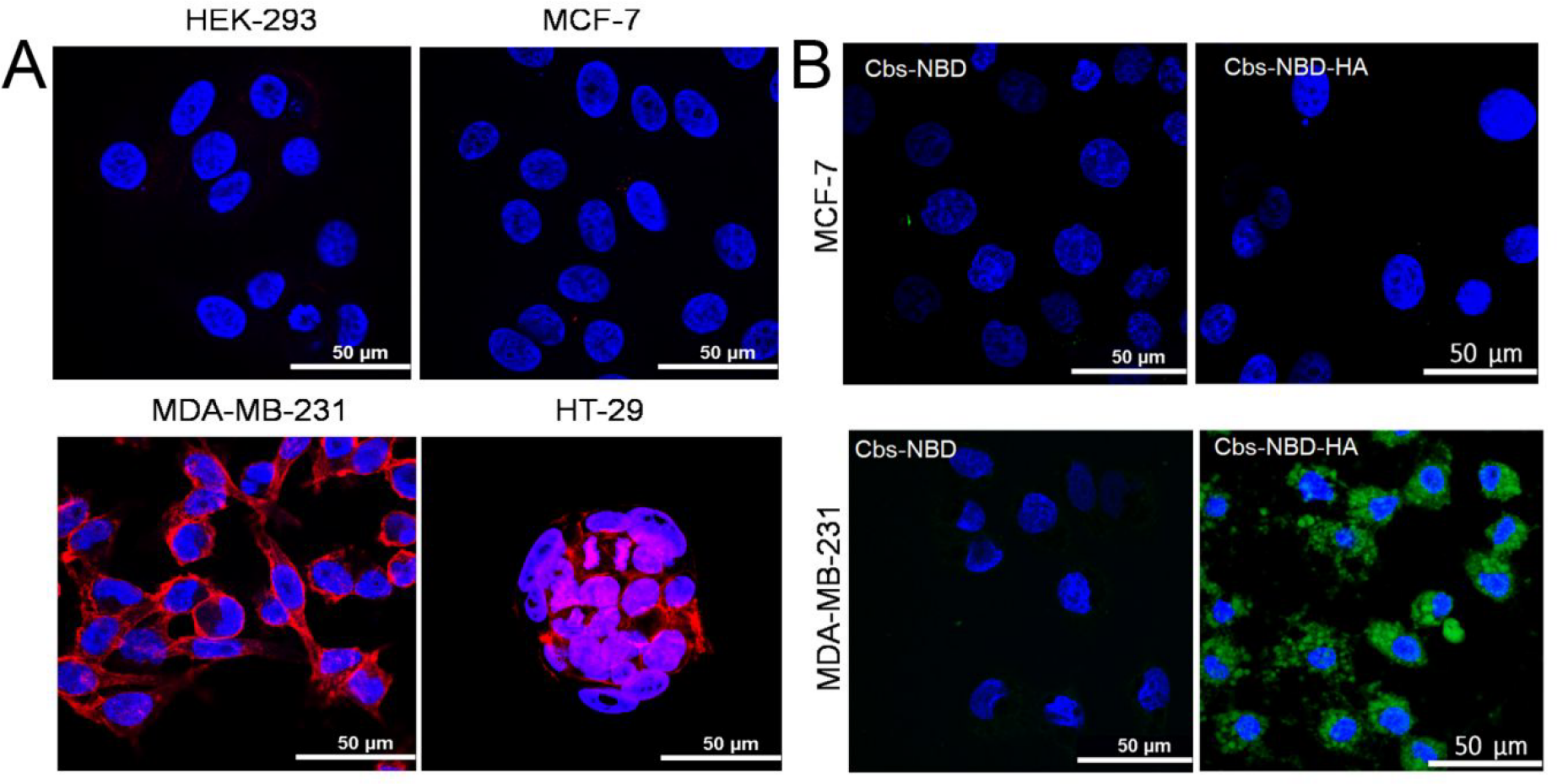
CD44 expression in cells allows targeting of cubosomes to cells with HA. (A) CD44 expression was studied in four different cell lines, as indicated, using immunofluorescence; red fluorescence indicates CD44 expression, while blue represents a nuclear counterstain (DAPI). (B) CD44-positive MDA-MB-231 cells or CD44-negative MCF-7 cells were treated with 20 μg/mL of fluorescently labeled (NBD) cubosomes either with (Cbs-NBD-HA) or without (Cbs-NBD) the HA ligand for 24 h under standard culture conditions. Uptake and localization were assessed using confocal microscopy with consistent settings to allow comparisons; green fluorescence indicates the presence of NBD dye, while blue represents a nuclear counterstain (DAPI).

CD44 is a cell surface adhesion receptor that binds the extracellular ligand hyaluronic acid (HA). CD44 is overexpressed in a range of cancers including colon, lung, ovarian, and triple-negative breast cancer.^13,30^ Additionally, CD44 has been identified as a marker for breast cancer stem-like cells, a subpopulation of cancer cells that are strongly linked to metastases, drug resistance, relapses following therapy, and poor clinical outcomes.^13^ We selected four cell lines, in which we assessed CD44 expression and its potential for HA-targeted delivery; one was a noncancerous line (HEK-293),^31^ one was a breast cancer line that reportedly does not express CD44 (MCF-7),^32,33^ and two were cancer lines that reportedly express CD44 (MDA-MB-231 and HT29, derived from triple-negative breast cancer and colon cancer, respectively).^32−35^ Using immunofluorescence, we confirmed that MDA-MB-231 and HT29 cells express dramatically more CD44 than the other lines, with MDA-MB-231 showing prominent expression on every cell, whereas HT29 showed more variable expression mainly in areas of cell-to-cell contact (Figure 2A).

HA has been used in preclinical therapeutic applications for targeting cancer cells through its function as a CD44 ligand, including in TNBC^36^ and colon cancer.^37^ In order to evaluate the binding specificity of HA-tagged cubosomes, the HA-tagged cubosomes (Cbs-NBD-HA) were labeled with 0.5% w/w of the fluorescent lipid 1,2-dioleoyl-*sn*-glycero-3-phosphoethanolamine-*N*-(7-nitro-2–1,3-benzoxadiazol-4-yl) (ammonium salt) (NBD-PE), and its localization was studied in two of the cell lines, including one that was CD44-negative (MCF-7) and one that was CD44-positive (MDA-MB-231). The CD44-positive line (MDA-MB-231), but not the negative line (MCF7), 24 h post treatment, showed specific uptake of fluorescent cubosomes as observed by confocal microscopy (Figure 2B), demonstrating dependence on cellular expression of CD44. We also demonstrated that uptake was dependent on the HA ligand, as fluorescent cubosomes lacking this targeting agent were not taken up by either MDA-MB-231 or MCF-7 cells (Figure 2B). Prange et al. have previously demonstrated that cubosome uptake by cells occurs by endocytosis;^38^ we infer that HA and CD44 combine here to induce the same uptake in MDA-MB-231 cells as punctate spots of green fluorescence are found inside the cells, although we have not formally proved involvement of the endocytic pathway. These data suggest that HA-tagged cubosomes could selectively deliver a drug payload to CD44-expressing cancer cells.

### CuAc-Loaded, HA-Tagged Cubosomes Kill CD44-Positive Cancer Cells

**Figure 3:**
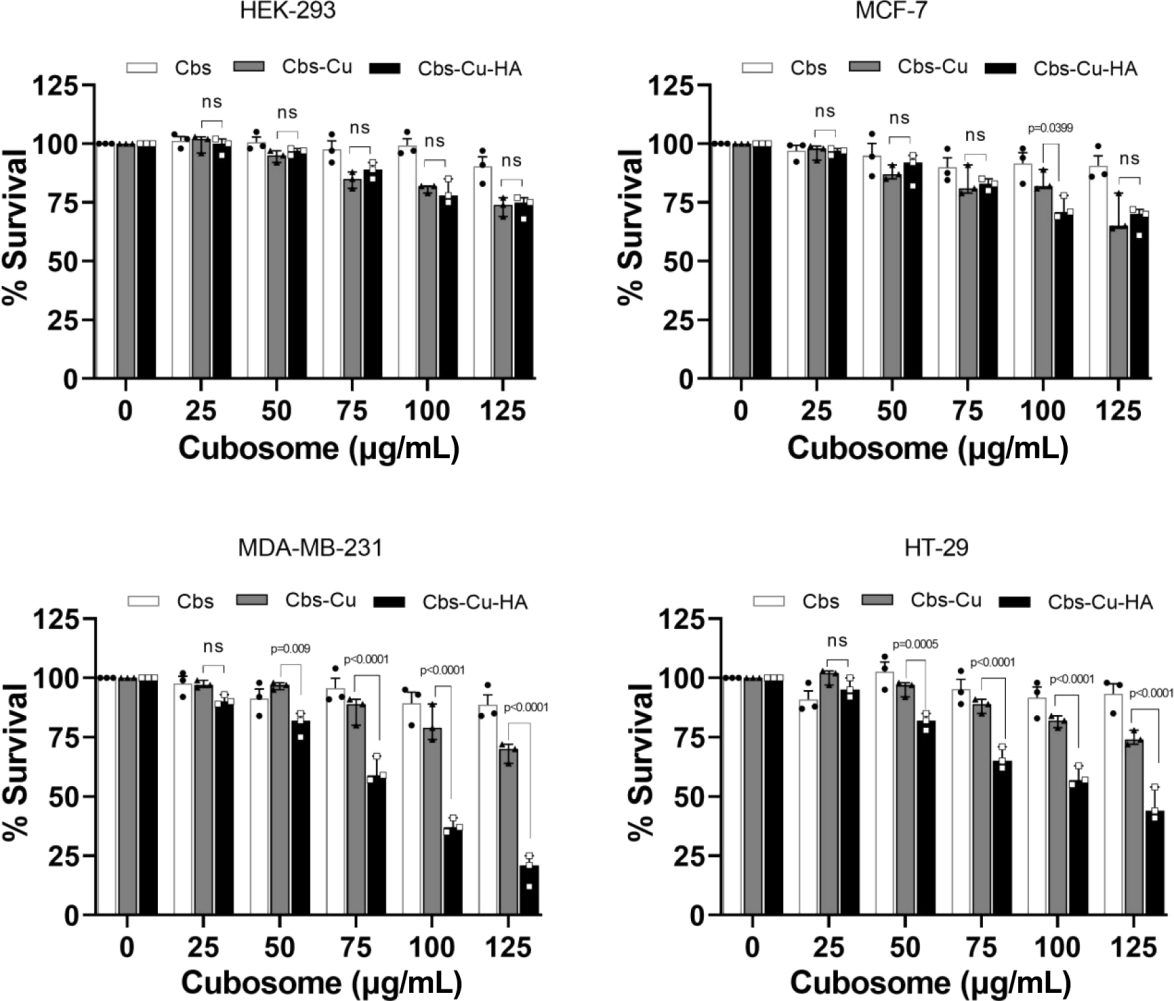
CuAc-loaded, HA-tagged cubosomes specifically kill CD44-positive cancer cell lines. Cell lines as indicated were treated with the doses shown of bare cubosomes (Cbs), drug-loaded cubosomes (Cbs-Cu), or HA-tagged, drug-loaded cubosomes (Cbs-Cu-HA) for 24 h. Survival of viable cells was assessed using MTT assays. Data represents means and standard errors of three independent experiments. Statistical analyses: two way ANOVA. ns: not significant.

Next, we assessed targeted delivery and efficacy of the potential cancer drug CuAc in HA-tagged cubosomes using *in vitro* cytotoxicity MTT assays. All four cell lines, including those that are CD44-positive and negative, were treated with a range of concentrations (0–125 μg/mL) of drug-loaded untargeted cubosomes (Cbs-Cu), or drug-loaded HA-tagged cubosomes (Cbs-Cu-HA), and cell survival was assessed (Figure 3). There are several reports demonstrating relatively low toxicity of MO-based cubosomes;^39^ however, as our formulation is developed from a novel ratio of MO:DPZ:F127, we also evaluated the toxicity of untargeted cubosomes without drug (Cbs). It was observed that bare cubosome (Cbs) did not show any significant cytotoxicity at any dose in any of the cell lines (Figure 3). Untargeted drug-loaded cubosomes (Cbs-Cu) showed some cytotoxicity at the highest concentrations (up to 25% cytotoxicity at 125 μg/mL), reflecting nonspecific uptake of the cubosomes and the payload drug, although at all lower concentrations, this cytotoxicity was not significantly different from the cubosomes lacking drug. Most notably, Cbs-Cu-HA showed a substantial and significant reduction in cell viability in the CD44-expressing cell lines while causing lesser degrees of cytotoxicity in the CD44-negative lines that were similar to toxicity caused by untargeted particles (Cbs-Cu). For example, at 75 and 100 μg/mL doses, the targeted cubosomes reduced viability by 41 and 63% in the CD44-positive cells MDA-MB-231, as compared to 11 and 22% for the untargeted cubosomes (*p* = 0.001).

Similarly, in HT-29 cells, which also overexpress CD44, the targeted formulation reduced viability 35% at 75 μg/mL and 43% at 100 μg/mL, compared to 12 and 18% viability reduction with the untargeted cubosomes (*p* = 0.001). These finding correlates with the *in vitro* localization data (Figure 2B), as CD44-expressing cells selectively uptake the targeted cubosomes, thereby receiving high levels of cytotoxic drug causing toxicity. Importantly, we also assessed the relative sensitivities of these cell lines to native, unencapsulated CuAc (Figure S6); this demonstrated no intrinsic significant differences in sensitivity to the drug between the cell lines, with MDA-MB-231 demonstrating the numerically highest IC50 indicative of greatest resistance. This confirmed that differential sensitivity to Cbs-Cu-HA related to targeting to the expression of CD44, rather than relating to intrinsic CuAc sensitivities.

### CuAc-Loaded, HA-Tagged Cubosomes Induce Apoptotic Death in 3D Spheroid Models

**Figure 4:**
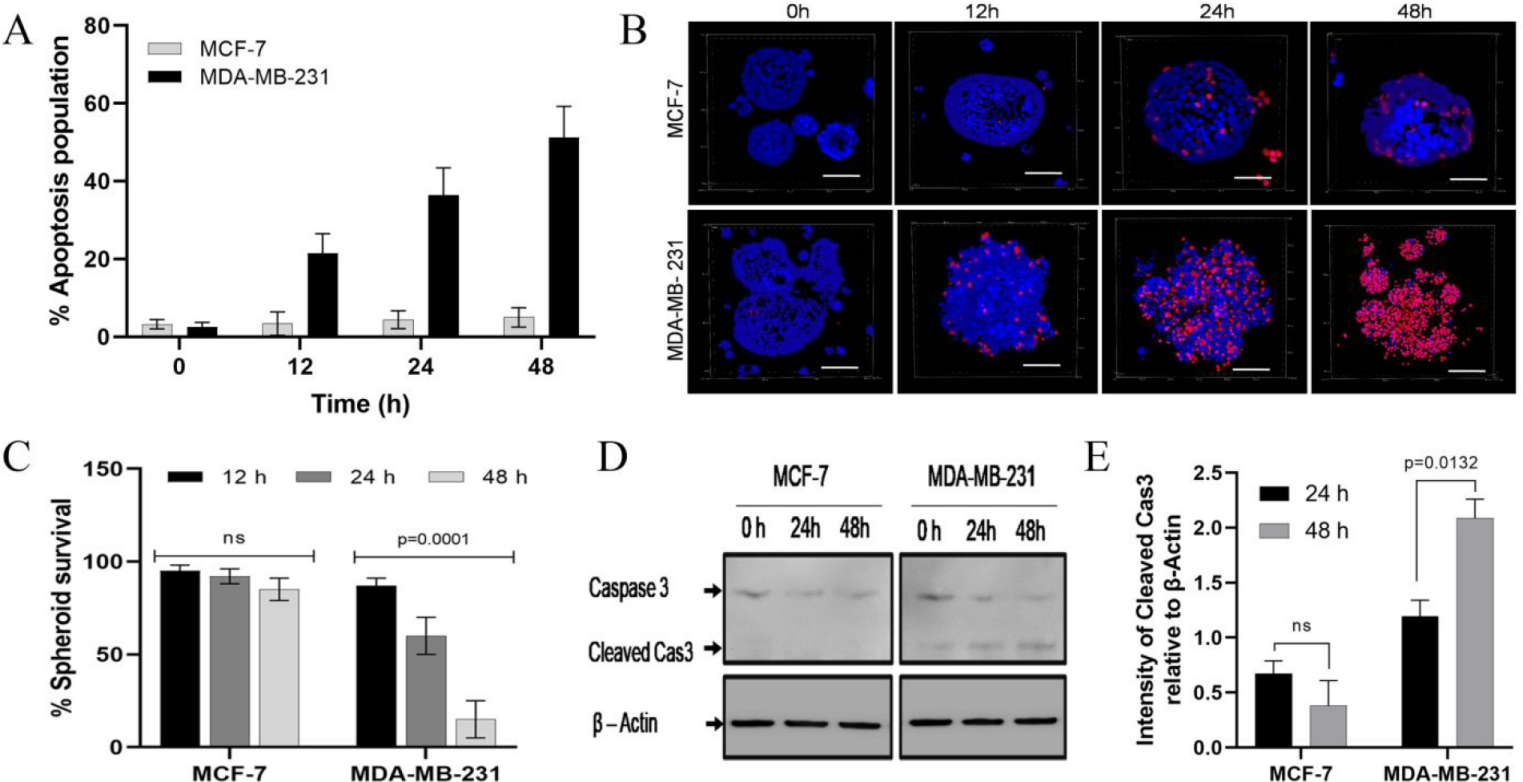
CuAc-loaded, HA-tagged cubosomes induce apoptosis in CD44-positive cells in 2D and 3D cultures. (A) MCF-7 or MDA-MB-231 cells in 2D culture were treated with 75 μg/mL Cbs-Cu-HA for up to 48 h; apoptosis was quantified using Annexin V/PI staining at the time points shown. (B,C) MCF-7 or MDA-MB-231 spheroids were established, which were then treated with 75 μg/mL Cbs-Cu-HA for up to 48 h; cell death was visualized (B; scale bar 200 μm) and quantified (C) using staining with PI and Hoechst 33342 and fluorescence microscopy and counting of the proportion of PI-positive cells. (D,E) MCF-7 or MDA-MB-231 spheroids were established and treated as for panels B and C; spheroids were lysed at the times indicated, and apoptosis was assessed by Western blotting for caspase 3 relative to the loading control beta-actin. A representative blot is shown (D) along with densitometry of cleaved caspase 3 (E). Quantitative data represents means and standard errors of three independent experiments, and statistical analyses were performed using two way ANOVA tests.

After confirming active targeting in both CD44-expressing cells in our panel of four lines, we further studied only the lines of breast cancer origin; CD44-positive MDA-MB-231 and CD44-negative MCF-7. Next, we assessed the mode of cell death, i.e., apoptosis or necrosis, using annexin V assays. Cells were treated with 75 μg/mL Cbs-Cu-HA; this dose was selected from data in Figure 3 as showing strong, targeted (HA-dependent) cytotoxicity in CD44-positive cells. Cells showing apoptosis were quantified before treatment (0 h) and at time points up to 48 h after treatment. MCF-7 cells showed negligible apoptosis, with only a small increase in the percentage of apoptotic cells from 3.2% before treatment to 5.6% after 48 h of treatment (Figure 4A). On the contrary, CD44-positive MDA-MB-231 cells showed time-dependent induction of apoptosis with up to 51% apoptosis at 48 h (Figure 4A). Thus, it was confirmed that targeted delivery of CuAc via Cbs-HA resulted in selective apoptosis of CD44-expressing cells.

Monolayer cultures do not mimic the structure and drug resistance conferred by elements of tumor microenvironment,^40^ whereas spheroids more closely resemble some aspects of the three-dimensional environment of cancer cells and thus offer better opportunities to study cancer drug behavior.^41^ We further evaluated our targeted particles (Cbs-Cu-HA) for efficacy in 3D spheroids of MDA-MB-231 and MCF-7. Spheroids were treated with 75 μg/mL of Cbs-Cu-HA for various time points up to 48 h. Using propidium iodide to assay cellular viability (viable cells exclude this dye) along with Hoechst 33342 (which permeates both viable and nonviable cells),^42^ we noted viability was reduced to 60% after 24 h and further to 10% (*p* = 0.0001) after 48 h of treatment in the case of the MDA-MB-231 spheroid, while only minor and nonsignificant reductions in viability were seen with the MCF-7 spheroids (Figure 4B,C). Interestingly, there was little evidence of the center of spheroids being relatively resistant, which can occur due to poor penetration of the cytotoxic, suggesting that Cbs-Cu-HA had access to the spheroid interior. Thus, we concluded that HA-tagged cubosomes could efficiently and selectively eliminate CD44-expressing cancer cells even in 3D culture conditions, without causing toxicity to cells lacking CD44. Apoptosis was observed to be the mode of cell death in the 2D cell culture study, so we further aimed to confirm the same in the 3D model. The presence of cleaved caspase 3, a common marker for apoptosis in spheroids,^43^ was used to assess apoptosis in protein extracts from spheroids before treatment or those treated with Cbs-Cu-HA at 24 or 48 h. As noted in Figure 4D, MDA-MB-231 spheroids showed caspase 3 cleavage at 24 h, which further increased at 48 h. MCF-7 showed no sign of caspase 3 cleavage even at 48 h. Thus, it was confirmed that the targeted delivery could successfully induce selective apoptosis and potentially eliminate CD44-expressing cancer cells.

### *In Vivo* Biocompatibility

**Figure 5:**
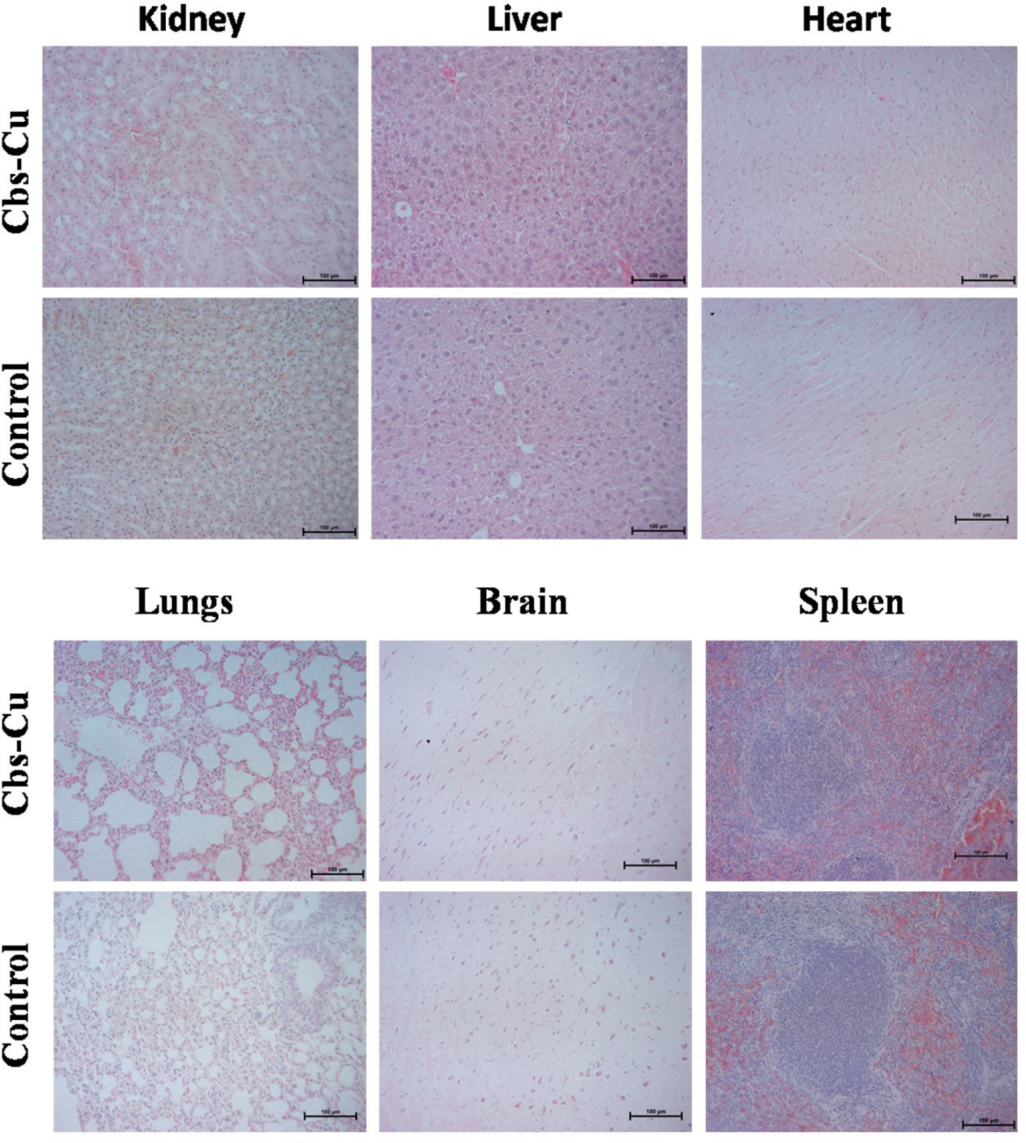
CuAc-loaded cubosomes do not cause tissue damage in critical organs. SCID mice were treated intravenously with repeated doses of Cbs-Cu over 6 days or with saline (control). Six days later, tissues were harvested, and sections were stained using hematoxylin and eosin to assess whether tissue damage was evident.

As a preliminary evaluation of toxicity of the formulated Cbs-Cu-HA and its future *in vivo* applicability, we studied the toxicity of Cbs-Cu in mice. A total of 12 nude mice were randomly divided into 2 groups, 1 of which served as control (injected with saline) and the other was administered with 100 μL of Cbs-Cu in PBS (18 mg/mL). These treatments were repeated 2 days later and again after a further 2 days. Tissue histology is commonly used to analyze *in vivo* toxicity for drug trials.^8,30^ Five days after the third treatment, tissue sections of organs including lung, brain, spleen, kidney, liver, and heart were studied using hematoxylin and eosin (H&E) staining. As observed in Figure 5, Cbs-Cu treatment was associated with no obvious toxicity as indicated by the absence of any necrotic tissue or abnormalities in any of these organs when compared with control group tissues. Change in total body during treatment indicates the health of mice and therefore can be used to monitor toxicity.^44^ In our case, we did not observe any significant change in the treated group with respect to control, which further indicates absence of any toxicity from Cbs-Cu treatment (Figure S7). Thus, we could conclude that Cbs-Cu poses little risk of toxicity in normal tissues for *in vivo* applications and therefore that use of these particles appears to be a viable strategy for targeting cancers. Similarly, others have demonstrated low toxicity in normal tissues *in vivo* to be associated with use of HA-targeted nanoformulations, thereby supporting the viability of our Cbs-Cu-HA particles as a cancer therapy.^45,46^

## Conclusion

We report a formulation of clickable cubosome nanoparticles based on monoolein with an internal nanostructure belonging to space group Im3m. The advantage of the clickable chemistry is its ease of functionalization with any ligand, particularly for cancer targeting applications. This is the first report of cubosomes tagged with hyaluronic acid using copper free click chemistry in order to target CD44-expressing cancer cells. We demonstrate this targeting and its specificity in monolayer cell culture and in 3D tumor spheroid models. The model drug used in this study, copper acetylacetonate, effectively killed cancer cells, at least in part by inducing apoptosis, when delivered via this targeted nanocarrier. Other groups have successfully encapsulated different cancer drugs into related cubosome formulations; examples include cisplatin and paclitaxel,^47^ SN38,^48^ or methotrexate.^49^ Therefore, we expect our targeting methodology to have broad applicability. Our preliminary *in vivo* study confirmed that the nanoformulation is nontoxic to normal tissues. There are currently very few published targeted drug delivery studies using cubosomes in the cancer field, and this work represents a major advance with potential clinical utility.

## Supporting Information Available

Figures S1 (infrared spectra of cubosomes), S2 (dynamic light scattering data for cubosomes), S3 (EDAX spectrum for cubosomes), S4 (zeta potential measurements for cubosomes), S5 (TEM image of cubosomes), S6 (cytotoxicity data for CuAc on the four cell lines), and S7 (body weight data for the *in vivo* analyses) (PDF)

## References

[ref1] Ferlay J.; Colombet M.; Soerjomataram I.; Parkin D. M.; Piñeros M.; Znaor A.; Bray F. Cancer statistics for the year 2020: An overview. Int. J. Cancer 2021, 149 (4), 778–789. 10.1002/ijc.33588. 33818764

[ref2] Oostra D. R.; Macrae E. R. Role of trastuzumab emtansine in the treatment of HER2-positive breast cancer. Breast Cancer (Dove Med. Press) 2014, 6, 103–113. 25114588 10.2147/BCTT.S67297 PMC4112743

[ref3] Dallas N. A.; Xia L.; Fan F.; Gray M. J.; Gaur P.; van Buren G. 2nd; Samuel S.; Kim M. P.; Lim S. J.; Ellis L. M. Chemoresistant colorectal cancer cells, the cancer stem cell phenotype, and increased sensitivity to insulin-like growth factor-I receptor inhibition. Cancer Res. 2009, 69 (5), 1951–1957. 10.1158/0008-5472.CAN-08-2023. 19244128 PMC3198868

[ref4] Nguyen T.-H.; Hanley T.; Porter C. J. H.; Larson I.; Boyd B. J. Phytantriol and glyceryl monooleate cubic liquid crystalline phases as sustained-release oral drug delivery systems for poorly water-soluble drugs II. In-vivo evaluation. J. Pharm. Pharmacol. 2010, 62 (7), 856–865. 10.1211/jpp.62.07.0006. 20636873

[ref5] Karami Z.; Hamidi M. Cubosomes: remarkable drug delivery potential. Drug Discovery Today 2016, 21 (5), 789–801. 10.1016/j.drudis.2016.01.004. 26780385

[ref6] Barriga H. M. G.; Holme M. N.; Stevens M. M. Cubosomes: The Next Generation of Smart Lipid Nanoparticles?. Angew. Chem., Int. Ed. 2019, 58 (10), 2958–2978. 10.1002/anie.201804067. PMC6606436 29926520

[ref7] Tiernan J. P.; Ingram N.; Marston G.; Perry S. L.; Rushworth J. V.; Coletta P. L.; Millner P. A.; Jayne D. G.; Hughes T. A. CEA-targeted nanoparticles allow specific in vivo fluorescent imaging of colorectal cancer models. Nanomedicine 2015, 10 (8), 1223–1231. 10.2217/nnm.14.202. 25694062

[ref8] Pramanik A.; Xu Z.; Shamsuddin S. H.; Khaled Y. S.; Ingram N.; Maisey T.; Tomlinson D.; Coletta P. L.; Jayne D.; Hughes T. A.; Tyler A. I. I.; Millner P. A. Affimer Tagged Cubosomes: Targeting of Carcinoembryonic Antigen Expressing Colorectal Cancer Cells Using In Vitro and In Vivo Models. ACS Appl. Mater. Interfaces 2022, 14 (9), 11078–11091. 10.1021/acsami.1c21655. 35196008 PMC9007418

[ref9] Aleandri S.; Bandera D.; Mezzenga R.; Landau E. M. Biotinylated Cubosomes: A Versatile Tool for Active Targeting and Codelivery of Paclitaxel and a Fluorescein-Based Lipid Dye. Langmuir 2015, 31 (46), 12770–12776. 10.1021/acs.langmuir.5b03469. 26513646

[ref10] Caltagirone C.; Falchi A. M.; Lampis S.; Lippolis V.; Meli V.; Monduzzi M.; Prodi L.; Schmidt J.; Sgarzi M.; Talmon Y.; Bizzarri R.; Murgia S. Cancer-Cell-Targeted Theranostic Cubosomes. Langmuir 2014, 30 (21), 6228–6236. 10.1021/la501332u. 24815031

[ref11] Zhai J.; Luwor R. B.; Ahmed N.; Escalona R.; Tan F. H.; Fong C.; Ratcliffe J.; Scoble J. A.; Drummond C. J.; Tran N. Paclitaxel-Loaded Self-Assembled Lipid Nanoparticles as Targeted Drug Delivery Systems for the Treatment of Aggressive Ovarian Cancer. ACS Appl. Mater. Interfaces 2018, 10 (30), 25174–25185. 10.1021/acsami.8b08125. 29963859

[ref12] Seok H.-Y.; Sanoj Rejinold N.; Lekshmi K. M.; Cherukula K.; Park I.-K.; Kim Y.-C. CD44 targeting biocompatible and biodegradable hyaluronic acid cross-linked zein nanogels for curcumin delivery to cancer cells: In vitro and in vivo evaluation. J. Controlled Release 2018, 280, 20–30. 10.1016/j.jconrel.2018.04.050. 29723613

[ref13] Jin J.; Krishnamachary B.; Mironchik Y.; Kobayashi H.; Bhujwalla Z. M. Phototheranostics of CD44-positive cell populations in triple negative breast cancer. Sci. Rep. 2016, 6 (1), 27871 10.1038/srep27871. 27302409 PMC4908597

[ref14] Parayath N. N.; Parikh A.; Amiji M. M. Repolarization of Tumor-Associated Macrophages in a Genetically Engineered Nonsmall Cell Lung Cancer Model by Intraperitoneal Administration of Hyaluronic Acid-Based Nanoparticles Encapsulating MicroRNA-125b. Nano Lett. 2018, 18 (6), 3571–3579. 10.1021/acs.nanolett.8b00689. 29722542

[ref15] Bourguignon L. Y. W.; Xia W.; Wong G. Hyaluronan-mediated CD44 Interaction with p300 and SIRT1 Regulates β-Catenin Signaling and NFκB-specific Transcription Activity Leading to MDR1 and Bcl-xL Gene Expression and Chemoresistance in Breast Tumor Cells*. J. Biol. Chem. 2009, 284 (5), 2657–2671. 10.1074/jbc.M806708200. 19047049 PMC2631959

[ref16] Rao G.; Wang H.; Li B.; Huang L.; Xue D.; Wang X.; Jin H.; Wang J.; Zhu Y.; Lu Y.; Du L.; Chen Q. Reciprocal Interactions between Tumor-Associated Macrophages and CD44-Positive Cancer Cells via Osteopontin/CD44 Promote Tumorigenicity in Colorectal Cancer. Clin. Cancer Res. 2013, 19 (4), 785–797. 10.1158/1078-0432.CCR-12-2788. 23251004

[ref17] Chen C.; Zhao S.; Karnad A.; Freeman J. W. The biology and role of CD44 in cancer progression: therapeutic implications. Journal of Hematology & Oncology 2018, 11 (1), 64 10.1186/s13045-018-0605-5. 29747682 PMC5946470

[ref18] Filik J.; Ashton A. W.; Chang P. C. Y.; Chater P. A.; Day S. J.; Drakopoulos M.; Gerring M. W.; Hart M. L.; Magdysyuk O. V.; Michalik S.; Smith A.; Tang C. C.; Terrill N. J.; Wharmby M. T.; Wilhelm H. Processing two-dimensional X-ray diffraction and small-angle scattering data in DAWN 2. J. Appl. Crystallogr. 2017, 50 (3), 959–966. 10.1107/S1600576717004708. 28656043 PMC5458597

[ref19] Seddon J. M.; Squires A. M.; Conn C. E.; Ces O.; Heron A. J.; Mulet X.; Shearman G. C.; Templer R. H. Pressure-jump X-ray studies of liquid crystal transitions in lipids. Philosophical Transactions of the Royal Society A: Mathematical, Physical and Engineering Sciences 2006, 364 (1847), 2635–2655. 10.1098/rsta.2006.1844. 16973480

[ref20] Tang L.; Yang X.; Yin Q.; Cai K.; Wang H.; Chaudhury I.; Yao C.; Zhou Q.; Kwon M.; Hartman J. A.; Dobrucki I. T.; Dobrucki L. W.; Borst L. B.; Lezmi S.; Helferich W. G.; Ferguson A. L.; Fan T. M.; Cheng J. Investigating the optimal size of anticancer nanomedicine. Proc. Natl. Acad. Sci. U. S. A. 2014, 111 (43), 15344–15349. 10.1073/pnas.1411499111. 25316794 PMC4217425

[ref21] Sykes E. A.; Chen J.; Zheng G.; Chan W. C. W. Investigating the Impact of Nanoparticle Size on Active and Passive Tumor Targeting Efficiency. ACS Nano 2014, 8 (6), 5696–5706. 10.1021/nn500299p. 24821383

[ref22] Peng J. Q.; Fumoto S.; Suga T.; Miyamoto H.; Kuroda N.; Kawakami S.; Nishida K. Targeted co-delivery of protein and drug to a tumor in vivo by sophisticated RGD-modified lipid-calcium carbonate nanoparticles. J. Controlled Release 2019, 302, 42–53. 10.1016/j.jconrel.2019.03.021. 30926479

[ref23] Jia Y.-Y.; Zhang J.-J.; Zhang Y.-X.; Wang W.; Li C.; Zhou S.-Y.; Zhang B.-L. Construction of redox-responsive tumor targeted cisplatin nano-delivery system for effective cancer chemotherapy. Int. J. Pharm. 2020, 580, 119190 10.1016/j.ijpharm.2020.119190. 32151664

[ref24] Hoshyar N.; Gray S.; Han H.; Bao G. The effect of nanoparticle size on in vivo pharmacokinetics and cellular interaction. Nanomedicine (Lond) 2016, 11 (6), 673–692. 10.2217/nnm.16.5. 27003448 PMC5561790

[ref25] Tan C.-P.; Lu Y.-Y.; Ji L.-N.; Mao Z.-W. Metallomics insights into the programmed cell death induced by metal-based anticancer compounds. Metallomics 2014, 6 (5), 978–995. 10.1039/c3mt00225j. 24668273

[ref26] Zehra S.; Tabassum S.; Arjmand F. Biochemical pathways of copper complexes: progress over the past 5 years. Drug Discov Today 2021, 26 (4), 1086–1096. 10.1016/j.drudis.2021.01.015. 33486113

[ref27] Razis E.; Kassapian M.; Andriakopoulou C.; Martei Y. M.; Zurn S. J.; Hammad N.; Romero Y.; Dafni U.; Ilbawi A. M.; Trapani D. Essential medicines list in national cancer control plans: a secondary analysis from a global study. Lancet Oncol 2022, 23 (3), e144–154. 10.1016/S1470-2045(21)00706-3. 35240089

[ref28] Bazylińska U.; Kulbacka J.; Schmidt J.; Talmon Y.; Murgia S. Polymer-free cubosomes for simultaneous bioimaging and photodynamic action of photosensitizers in melanoma skin cancer cells. J. Colloid Interface Sci. 2018, 522, 163–173. 10.1016/j.jcis.2018.03.063. 29601958

[ref29] Pochapski D. J.; Carvalho dos Santos C.; Leite G. W.; Pulcinelli S. H.; Santilli C. V. Zeta Potential and Colloidal Stability Predictions for Inorganic Nanoparticle Dispersions: Effects of Experimental Conditions and Electrokinetic Models on the Interpretation of Results. Langmuir 2021, 37 (45), 13379–13389. 10.1021/acs.langmuir.1c02056. 34637312

[ref30] Gao Z.; Li Y.; Zhang Y.; An P.; Chen F.; Chen J.; You C.; Wang Z.; Sun B. A CD44-targeted Cu(ii) delivery 2D nanoplatform for sensitized disulfiram chemotherapy to triple-negative breast cancer. Nanoscale 2020, 12 (15), 8139–8146. 10.1039/D0NR00434K. 32236229

[ref31] Kawaguchi M.; Dashzeveg N.; Cao Y.; Jia Y.; Liu X.; Shen Y.; Liu H. Extracellular Domains I and II of cell-surface glycoprotein CD44 mediate its trans-homophilic dimerization and tumor cluster aggregation. J. Biol. Chem. 2020, 295 (9), 2640–2649. 10.1074/jbc.RA119.010252. 31969394 PMC7049959

[ref32] Li W.; Ma H.; Zhang J.; Zhu L.; Wang C.; Yang Y. Unraveling the roles of CD44/CD24 and ALDH1 as cancer stem cell markers in tumorigenesis and metastasis. Sci. Rep. 2017, 7 (1), 13856 10.1038/s41598-017-14364-2. 29062075 PMC5653849

[ref33] Smith S. M.; Cai L. Cell Specific CD44 Expression in Breast Cancer Requires the Interaction of AP-1 and NFκB with a Novel cis-Element. PLoS One 2012, 7 (11), e50867 10.1371/journal.pone.0050867. 23226410 PMC3511339

[ref34] Sahlberg S. H.; Spiegelberg D.; Glimelius B.; Stenerlöw B.; Nestor M. Evaluation of Cancer Stem Cell Markers CD133, CD44, CD24: Association with AKT Isoforms and Radiation Resistance in Colon Cancer Cells. PLoS One 2014, 9 (4), e94621 10.1371/journal.pone.0094621. 24760019 PMC3997403

[ref35] Mitchell B. S.; Whitehouse A.; Prehm P.; Delpech B.; Schumacher U. CD44 exon variant 6 epitope and hyaluronate synthase are expressed on HT29 human colorectal carcinoma cells in a SCID mouse model of metastasis formation. Clinical & Experimental Metastasis 1996, 14 (2), 107–114. 10.1007/BF00121207. 8605724

[ref36] Ben Daya S. M.; Paul V.; Awad N. S.; Al Sawaftah N. M.; Al Sayah M. H.; Husseini G. A. Targeting Breast Cancer Using Hyaluronic Acid-Conjugated Liposomes Triggered with Ultrasound. Journal of Biomedical Nanotechnology 2021, 17 (1), 90–99. 10.1166/jbn.2021.3012. 33653499

[ref37] Mansoori B.; Mohammadi A.; Abedi-Gaballu F.; Abbaspour S.; Ghasabi M.; Yekta R.; Shirjang S.; Dehghan G.; Hamblin M. R.; Baradaran B. Hyaluronic acid-decorated liposomal nanoparticles for targeted delivery of 5-fluorouracil into HT-29 colorectal cancer cells. Journal of Cellular Physiology 2020, 235 (10), 6817–6830. 10.1002/jcp.29576. 31989649 PMC7384933

[ref38] Prange J. A.; Aleandri S.; Komisarski M.; Luciani A.; Käch A.; Schuh C.-D.; Hall A. M.; Mezzenga R.; Devuyst O.; Landau E. M. Overcoming Endocytosis Deficiency by Cubosome Nanocarriers. ACS Applied Bio Materials 2019, 2 (6), 2490–2499. 10.1021/acsabm.9b00187. 35030705

[ref39] Zhai J.; Tan F. H.; Luwor R. B.; Srinivasa Reddy T.; Ahmed N.; Drummond C. J.; Tran N. In Vitro and In Vivo Toxicity and Biodistribution of Paclitaxel-Loaded Cubosomes as a Drug Delivery Nanocarrier: A Case Study Using an A431 Skin Cancer Xenograft Model. ACS Applied Bio Materials 2020, 3 (7), 4198–4207. 10.1021/acsabm.0c00269. 35025421

[ref40] Breslin S.; O’Driscoll L. Three-dimensional cell culture: the missing link in drug discovery. Drug Discovery Today 2013, 18 (5), 240–249. 10.1016/j.drudis.2012.10.003. 23073387

[ref41] Han S. J.; Kwon S.; Kim K. S. Challenges of applying multicellular tumor spheroids in preclinical phase. Cancer Cell International 2021, 21 (1), 152 10.1186/s12935-021-01853-8. 33663530 PMC7934264

[ref42] Brüningk S. C.; Rivens I.; Box C.; Oelfke U.; ter Haar G. 3D tumour spheroids for the prediction of the effects of radiation and hyperthermia treatments. Sci. Rep. 2020, 10 (1), 1653 10.1038/s41598-020-58569-4. 32015396 PMC6997397

[ref43] Ngaokrajang U.; Janvilisri T.; Sae-Ueng U.; Prungsak A.; Kiatwuthinon P. Integrin α5 mediates intrinsic cisplatin resistance in three-dimensional nasopharyngeal carcinoma spheroids via the inhibition of phosphorylated ERK /caspase-3 induced apoptosis. Exp. Cell Res. 2021, 406 (2), 112765 10.1016/j.yexcr.2021.112765. 34358523

[ref44] Zhao H.; Feng H.; Liu D.; Liu J.; Ji N.; Chen F.; Luo X.; Zhou Y.; Dan H.; Zeng X.; Li J.; Sun C.; Meng J.; Ju X.; Zhou M.; Yang H.; Li L.; Liang X.; Chu L.; Jiang L.; He Y.; Chen Q. Self-Assembling Monomeric Nucleoside Molecular Nanoparticles Loaded with 5-FU Enhancing Therapeutic Efficacy against Oral Cancer. ACS Nano 2015, 9 (10), 9638–9651. 10.1021/acsnano.5b04520. 26349079

[ref45] Dong S.; Bi Y.; Sun X.; Zhao Y.; Sun R.; Hao F.; Sun Y.; Wang Y.; Li X.; Deng W.; Liu X.; Ha J.; Teng L.; Gong P.; Xie J.; Kim B. Y. S.; Yang Z.; Jiang W.; Teng L. Dual-Loaded Liposomes Tagged with Hyaluronic Acid Have Synergistic Effects in Triple-Negative Breast Cancer. Small 2022, 18 (16), 2107690 10.1002/smll.202107690. PMC11878215 35277914

[ref46] Sun Y.; Li X.; Zhang L.; Liu X.; Jiang B.; Long Z.; Jiang Y. Cell Permeable NBD Peptide-Modified Liposomes by Hyaluronic Acid Coating for the Synergistic Targeted Therapy of Metastatic Inflammatory Breast Cancer. Mol. Pharmaceutics 2019, 16 (3), 1140–1155. 10.1021/acs.molpharmaceut.8b01123. 30668131

[ref47] Zhang L.; Li J.; Tian D.; Sun L.; Wang X.; Tian M. Theranostic combinatorial drug-loaded coated cubosomes for enhanced targeting and efficacy against cancer cells. Cell death & disease 2020, 11 (1), 1 10.1038/s41419-019-2182-0. 31911576 PMC6946659

[ref48] Ranneh A. H.; Iwao Y.; Noguchi S.; Oka T.; Itai S. The use of surfactants to enhance the solubility and stability of the water-insoluble anticancer drug SN38 into liquid crystalline phase nanoparticles. Int. J. Pharm. 2016, 515 (1–2), 501–505. 10.1016/j.ijpharm.2016.10.058. 27793711

[ref49] Mierzwa M.; Cytryniak A.; Krysiński P.; Bilewicz R. Lipidic Liquid Crystalline Cubic Phases and Magnetocubosomes as Methotrexate Carriers. Nanomaterials (Basel) 2019, 9 (4), 636 10.3390/nano9040636. 31010165 PMC6524136

