# Supplementary material for: Hyaluronic-Acid-Tagged Cubosomes Deliver Cytotoxics Specifically to CD44-Positive Cancer Cells
Source: Mol Pharm. 2022 Aug 8;19(12):4601–11. doi: 10.1021/acs.molpharmaceut.2c00439 (PMC9727730; doi:10.1021/acs.molpharmaceut.2c00439)
Supplement: Supplementary file 1 — mp2c00439_si_001.pdf [file mp2c00439_si_001.pdf]

Supplementary Information

Hyaluronic acid-tagged cubosomes deliver cytotoxics specifically to CD44-positive cancer cells

Arindam Pramanik[1*], Zexi Xu[2], Nicola Ingram[1], Patricia Louise Coletta[1], Paul A Millner[3], Arwen I.I. Tyler[2*], Thomas A Hughes[1*]

[1]School of Medicine, University of Leeds, Leeds, United Kingdom
[2]School of Food Science and Nutrition, University of Leeds, Leeds, United Kingdom
[3]School of Biomedical Sciences, University of Leeds, United Kingdom

Corresponding authors email addresses: a.pramanik@leeds.ac.uk; a.i.i.tyler@leeds.ac.uk; t.hughes@leeds.ac.uk

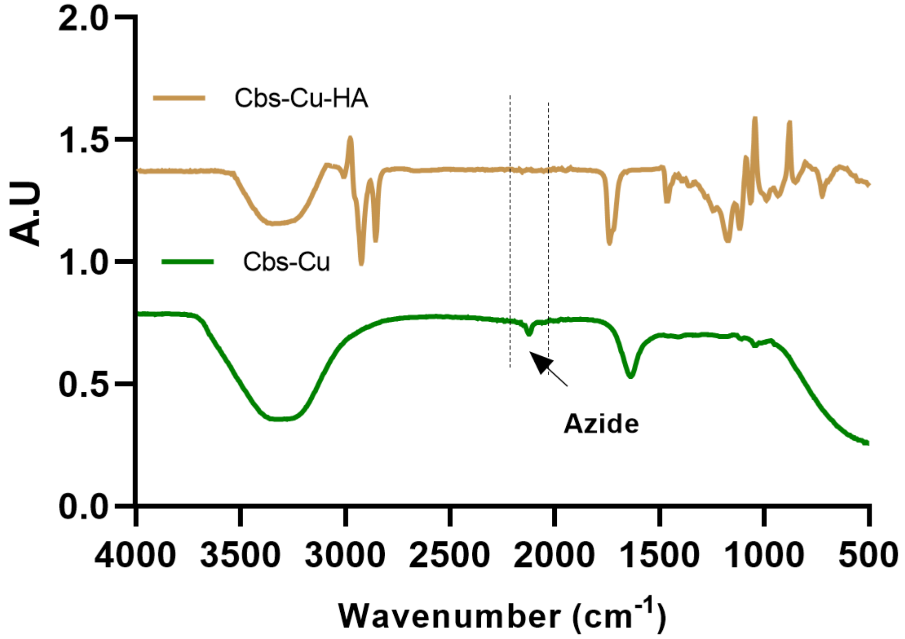

Figure S1. Fourier-transformed infrared spectra of HA tagged (Cbs-Cu-HA) and untagged (Cbs-Cu) cubosomes loaded with CuAc show that Cbs-Cu shows a peak at 2127 cm^{-1} attributed to the azide (N=N=N) functional group. After conjugation of HA-DBCO to the DSPE-azide group on the cubosomes, the peak is absent in the spectrum for Cbs-Cu-HA which confirmes the covalent linkage of HA to the cubosome surface.

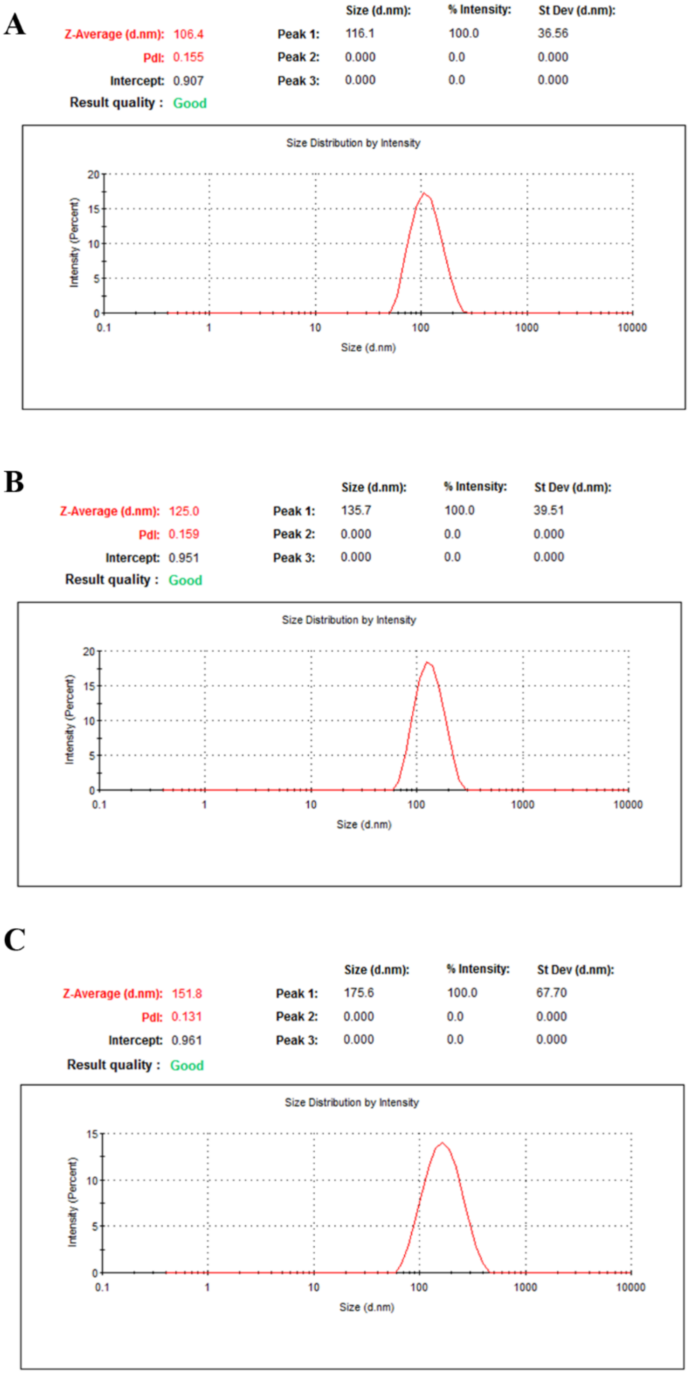

Figure S2. Dynamic light scattering data of bare cubosomes (A), CuAc-loaded cubosomes (B) and CuAc-loaded, HA-tagged cubosomes (C).

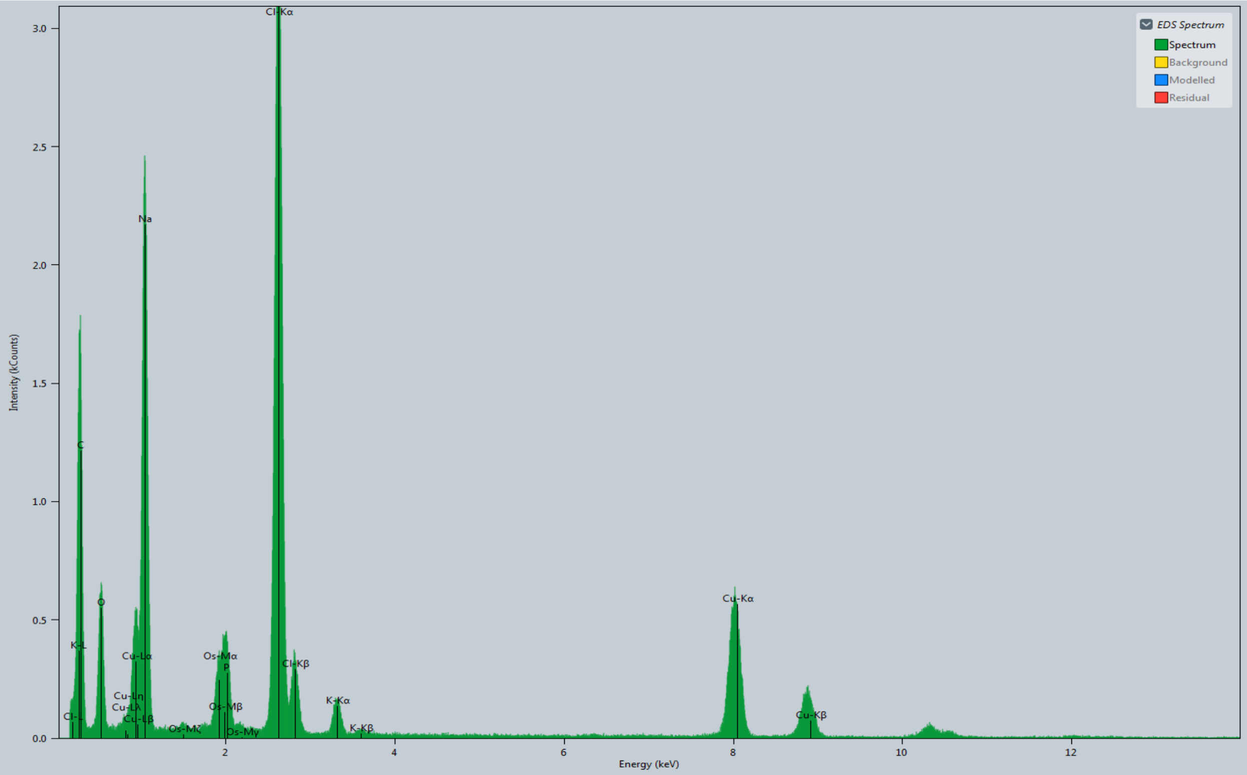

Figure S3. Energy Dispersive X-Ray (EDAX) spectrum of bare cubosomes (Cbs) showing very low intensity peaks for Copper compared to Cbs-Cu-HA.

Zeta Potential (mV):	-40.8
Zeta Deviation (mV):	7.73
Conductivity (mS/cm):	0.111
Result quality :	Good

	Mean (mV)	Area (%)	St Dev (mV)
Peak 1:	-40.8	100.0	7.73
Peak 2:	0.00	0.0	0.00
Peak 3:	0.00	0.0	0.00

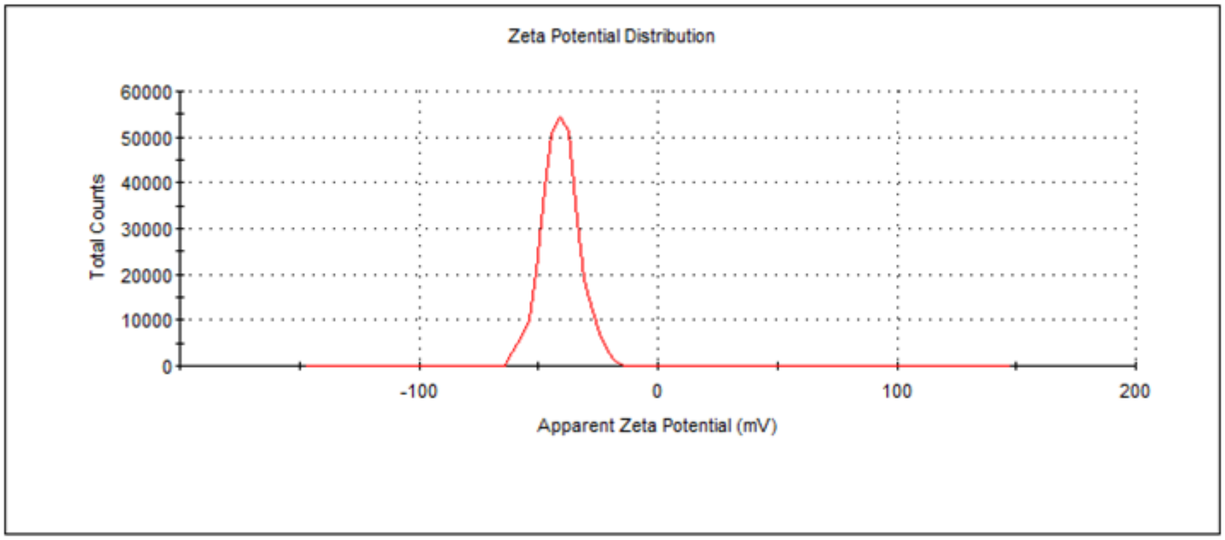

Figure S4. Zeta potential measurement. The surface charge of the Cbs-Cu-HA was found to be -40.8 mV which represents electrostatic stability of the nanoparticles.

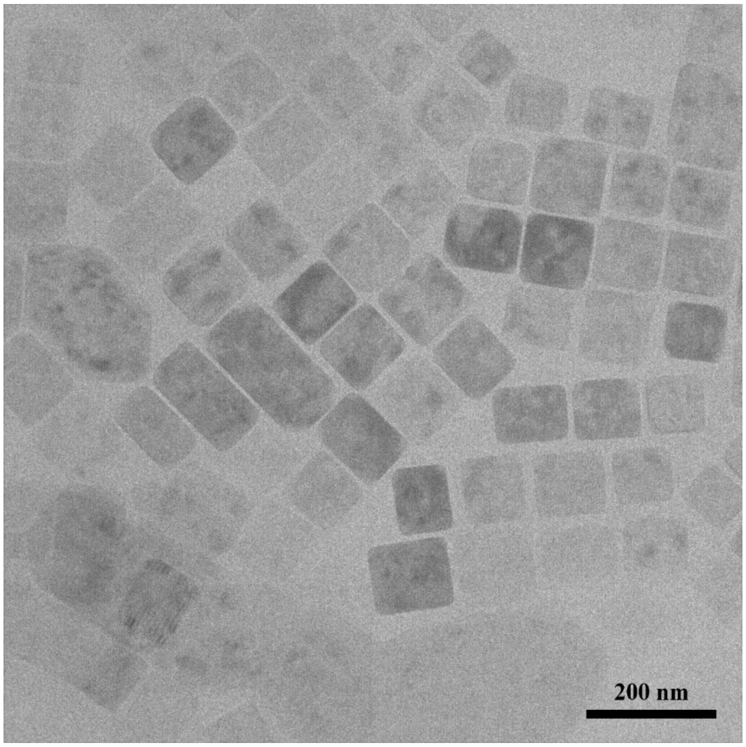

Figure S5. Transmission electron microscope (TEM) image for morphology and structure of the bare cubosome (Cbs).

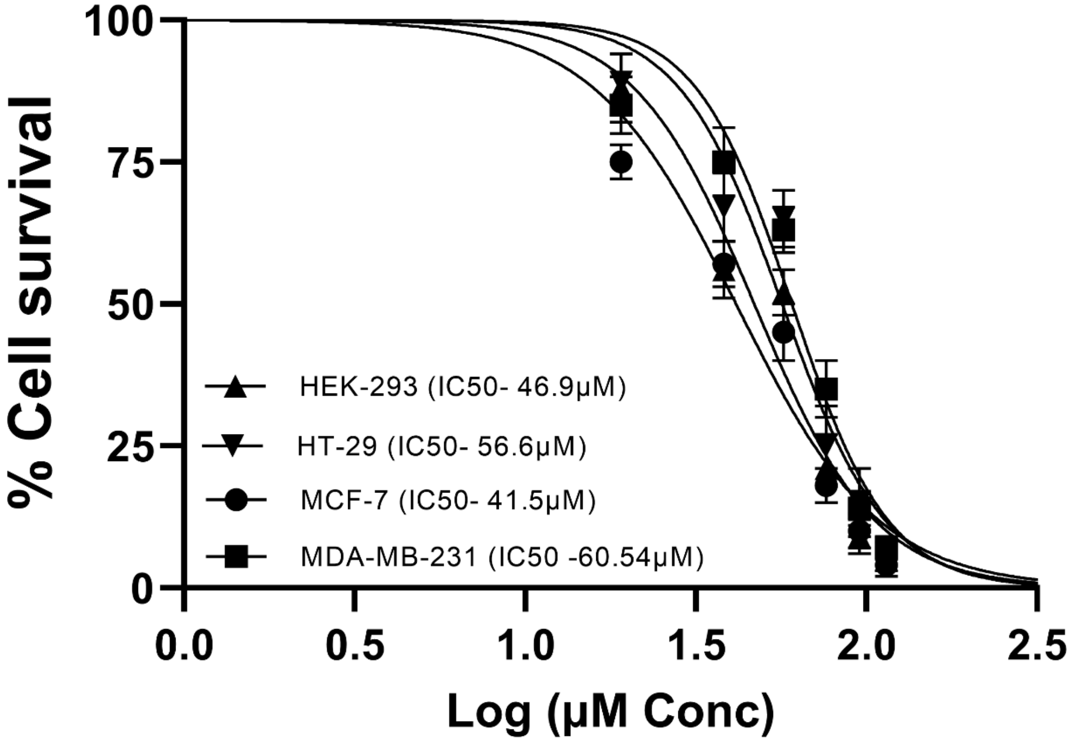

Figure S6. Cytotoxicity assay of CuAc on cells. MTT assay of unencapsulated CuAc showed dose dependent but non-selective cytotoxicity on all four cell lines with varying LD_{50} values (calculated from non-linear regression curves). Data show that CuAc itself does not have selectivity towards any cells and only when delivered via Cbs-HA could shows selective toxicity in CD44 expressing cells.

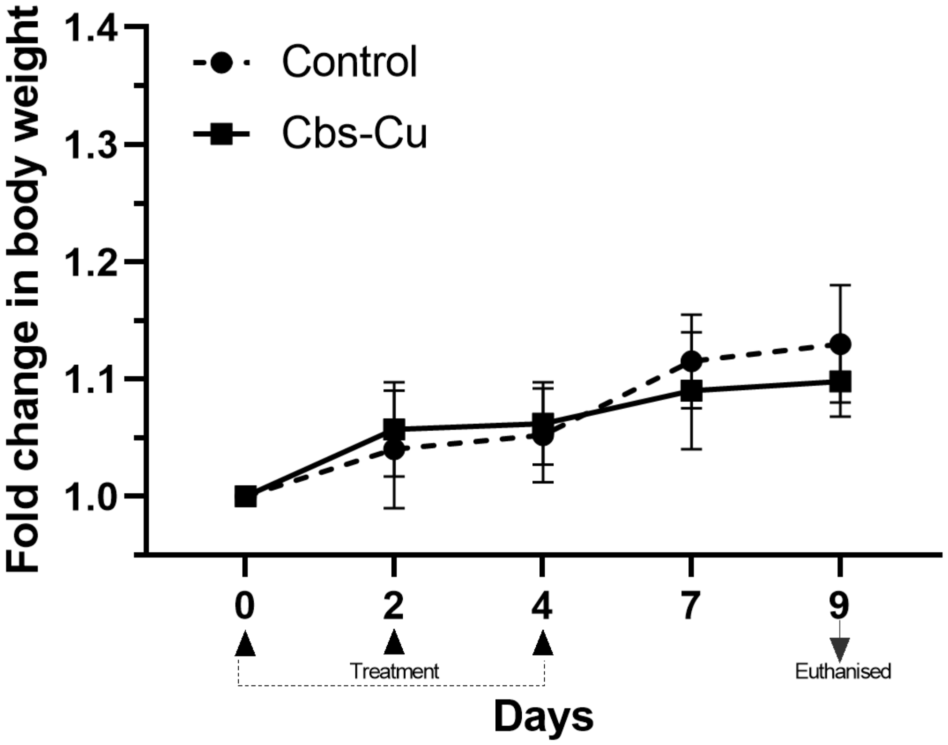

Figure S7. Fold change in mice body weight. The body weight of mice was noted for the two groups receiving either Cbs-Cu or saline (control) during the experiment conduct. There was no significant change or loss of body weight upon Cbs-Cu compared to control which indicates absence of toxicity.
